# Vision based interface system for hands free control of an intelligent wheelchair

**DOI:** 10.1186/1743-0003-6-33

**Published:** 2009-08-06

**Authors:** Jin Sun Ju, Yunhee Shin, Eun Yi Kim

**Affiliations:** 1 Visual Information Processing Labratory, Department of Advanced Technology Fusion, Konkuk, University, Seoul, South Korea

## Abstract

**Background:**

Due to the shift of the age structure in today's populations, the necessities for developing the devices or technologies to support them have been increasing. Traditionally, the wheelchair, including powered and manual ones, is the most popular and important rehabilitation/assistive device for the disabled and the elderly. However, it is still highly restricted especially for severely disabled. As a solution to this, the Intelligent Wheelchairs (IWs) have received considerable attention as mobility aids. The purpose of this work is to develop the IW interface for providing more convenient and efficient interface to the people the disability in their limbs.

**Methods:**

This paper proposes an intelligent wheelchair (IW) control system for the people with various disabilities. To facilitate a wide variety of user abilities, the proposed system involves the use of face-inclination and mouth-shape information, where the direction of an IW is determined by the inclination of the user's face, while proceeding and stopping are determined by the shapes of the user's mouth. Our system is composed of electric powered wheelchair, data acquisition board, ultrasonic/infra-red sensors, a PC camera, and vision system. Then the vision system to analyze user's gestures is performed by three stages: detector, recognizer, and converter. In the detector, the facial region of the intended user is first obtained using Adaboost, thereafter the mouth region is detected based on edge information. The extracted features are sent to the recognizer, which recognizes the face inclination and mouth shape using statistical analysis and *K*-means clustering, respectively. These recognition results are then delivered to the converter to control the wheelchair.

**Result & conclusion:**

The advantages of the proposed system include 1) accurate recognition of user's intention with minimal user motion and 2) robustness to a cluttered background and the time-varying illumination. To prove these advantages, the proposed system was tested with 34 users in indoor and outdoor environments and the results were compared with those of other systems, then the results showed that the proposed system has superior performance to other systems in terms of speed and accuracy. Therefore, it is proved that proposed system provided a friendly and convenient interface to the severely disabled people.

## Background

### Problem Statement

**Figure 1:**
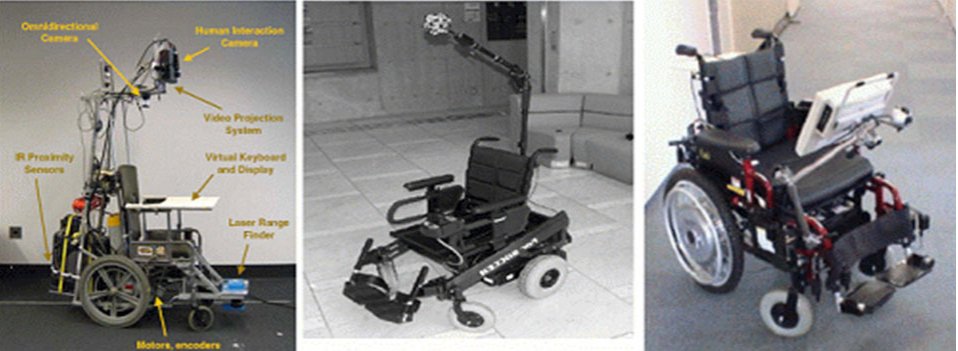
**Intelligent Wheelchairs (IWs)**. (a) GRASP Laboratory Smart Chair [6], (b) Wheelchair of Yutaka et. al [3], (c) Nav Chair [14].

With the increase of elderly and disabled people, a wide range support devices and care equipment has been developed to help improve their quality of life (QOL) [1,2]. In particular, intelligent wheelchairs (IWs) have received considerable attention as mobility aids. Essentially, IWs are electric powered wheelchairs (EPWs) with an embedded computer and sensors, giving them intelligence. Figure 1 shows the various IWs [3-9].

Two basic techniques have been required to develop IWs: 1) auto navigation techniques for automatic obstacle detection and avoidance, 2) convenient interfaces that allow handicapped users to control the IW themselves using their limited physical abilities. While it is important to develop a system that enables the user to assist in the navigation, the system is useless if it cannot be adapted to the abilities of the user. For example, in the case a user cannot manipulate a standard joystick, other control options need to be provided.

### Related Research

So far many access methods for IWs have been developed and then they can be classified as intrusive and non-intrusive. They are summarized in Table 1. Intrusive methods use glasses, a headband, or cap with infrared/ultrasound emitters to measure the user's intention based on changes in the ultrasound waves or infrared reflect [10-12]. In contrast, non-intrusive methods do not require any additional devices attached to user's face or head.

**Table 1:** IW controls in literatures

		**Intelligent Wheelchair**	**Feature**	**Device**	**Supporting Commands**
		
**Intrusive interfaces**		Y.L. Chen, et, al [10]	Head orientation	tilt sensors, microprocessor	Go, back, left, right
		
		SIAMO project [11]	Eye gaze	Electrode	Go, Back, Left, Right
		
		Wheelesley [12]	Eye gaze	Infrared sensors, ultrasonic range sensors, electrodes (EOG)	Go, Stop, Back, Left, Right
**Non-intrusive interfaces**	**voice**	Siamo project [11]	Voice	ultrasonic sensors, infrared sensors, camera & laser diode	Go, Back, Left, Right
		
		ROB Chair [13]	Voice	infrared sensors, ultrasonic sensors, head microphone	Go, Stop, Speed up, Speed Down, Rotate
		
		NAVChair [14]	Voice	Dos-based computer, ultrasonic transducer, lap tray, sonar sensors	Go, Stop, Back, Left, Right
		
		TAO project [15]	Voice	sensors, 2 processor boxes	Go, Stop, Back, Left, Right, Speed Down
	
	**vision**	Yoshida, et, al [22]	Face	ultrasonic sensors, 2 video camera	Go, Stop, Left, Right
		
		HGI [16]	Head & nose	webcam, ultrasonic sensors, data acquisition board	Go, Left, Right, Speed up, Speed Down
		
		SIAMO [11]	Head	CCD color-micro camera	Go, Left, Right, Speed up, Speed Down
		
		Proposed IW	Face & Mouth	web camera, data acquisition board	Single commands: Go, Stop, Left, Right, Rotate Mixing commands: Go-left, Go-Right

As shown in Table 1, voice-based and vision-based methods belong to the nonintrusive methods. Voice control is a natural and friendly access method, however, the existence of other noises in a real environment can lead to command recognition failure, resulting in safety problems [13-15]. Accordingly, a lot of research has been focused on vision-based interfaces, where control is derived from recognizing the user's gestures by processing images or videos obtained via a camera. With such interfaces, face or head movements are most widely used to convey the user's intentions. When a user wishes to move in a certain direction, it is a natural action to look in that direction, thus movement is initiated based on nodding the head, while turning is generated by the head direction. However, such systems have a major drawback, as they are unable to discriminate between intentional behavior and unintentional behavior. For example, it is natural for a user to look at an obstacle as it gets close, however, the system will turn and go towards that obstacle [16].

### Our Proposal

Accordingly, we develop a novel IW interface using face and mouth recognition for the severely disabled. The main goal of the present study is to provide a more convenient and effective access method for people with various disabilities. For accurate recognition of the user's intention, the direction of the IW is determined according to the face inclination, while proceeding and stopping are determined by the shape of the mouth. This format was inspired based on the operation of car, as the user's face movements correspond to the steering wheel, while the user's mouth corresponds to the brake and gas pedal. The mechanisms prevent an accident in the case the user instinctively turns their head to look at an obstacle, thereby making safer. Moreover, the proposed control mechanisms require minimal user motion, making the system more comfortable and more adaptable for the severely disabled when compared to conventional methods.

The proposed IW system consists of Facial Feature Detector (Detector), Facial Feature Recognizer (Recognizer), and Converter [17]. In our system, the facial region is first obtained using Adaboost algorithm, which is robust to the time-varying illumination [18,19]. Thereafter the mouth regions are detected based on edge information. These detection results are delivered to the Recognizer, which recognizes the face inclination and mouth shape. These recognition results are then delivered to the Converter, thereby the wheelchair are operated. To assess the effectiveness of the proposed interface, it was tested with 34 users and the results were compared with those of other systems. Then, the results showed that the proposed system has the superior performance to others in terms of accuracy and speed, and they also confirmed that the proposed system can accurately recognize user's gestures in real-time.

## Methods

### System Architecture

The proposed IW is composed of electric powered wheelchair, data acquisition board, and a PC camera and vision system. A data acquisition board (DAQ-board) is used to process the sensor information and control the wheelchair. The DAQ-board and a vision system are connected via a serial port. In our system, a FUJITSU (S6510) notebook is used as a vision system to process a video streaming received from a PC camera. The camera is connected to a vision system through a USB port and is mounted on the front of the wheelchair's tray, pointing down at an approximately 15 degree angle. The baseline between a user and camera is 25 cm (9.8 inches).

**Figure 2:**
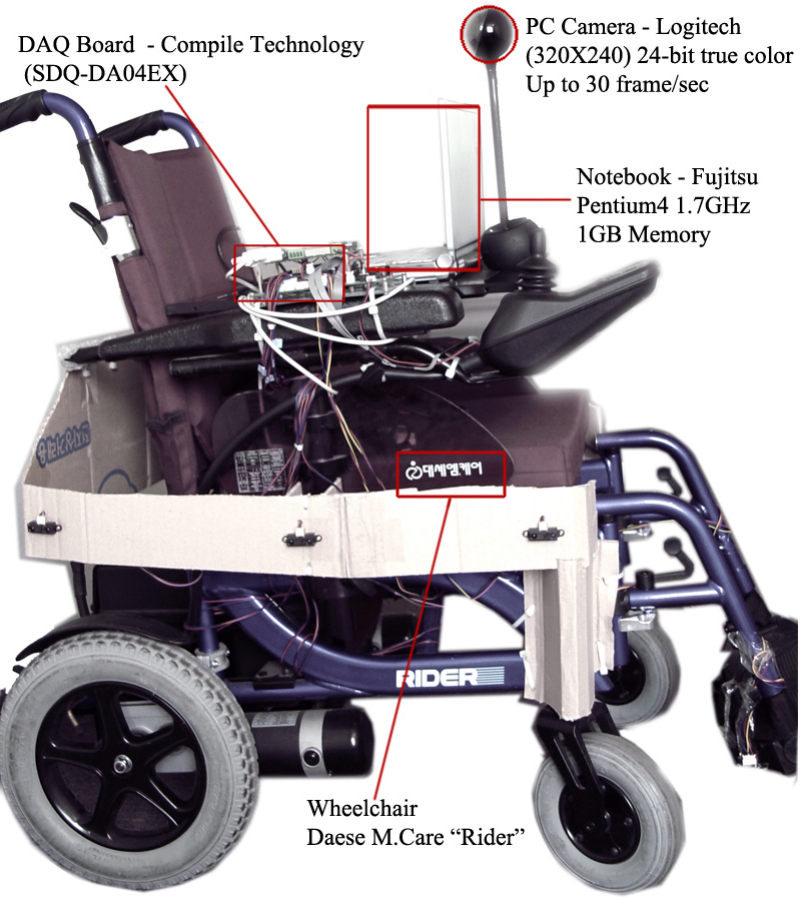
**The prototype of our IW**.

Our system is described in Figure 2 and specification of the components is illustrated in Table 2.

**Table 2:** The specification of the proposed IW

	**Hardware**		**Software**
Wheelchair	EPW-DAESE M. care Rider	OS	MS Window XP

DAQ Board	Compile Technology SDQ-DA04EX	Developed Language	MS Visual C++, MS Visual Basic 6.0

Input device	Logitech (640 × 480) Up to 30 frame/sec 24-Bit True Color	Camera Control	Open CV
		
Vision System	Pentium IV 1.7 GHz 1GB Memory		
		
Sensors	Two ultrasonic sensors Six Infra-red sensors		

### Overview of Vision-based Control System

The proposed control system receives and displays a live video streaming of the user sitting on the wheelchair in front of the computer. Then, the proposed interface allows the user to control the wheelchair directly by changing their face inclination and mouth shape. If the user wants the wheelchair to move forward, they just say "Go." Conversely, to stop the wheelchair, the user just says "Uhm." Here, the control commands using the shape of the mouth are only effective when the user is looking forward, thereby preventing over-recognition when the user is talking to someone. Meanwhile, the direction of the IW is determined by the inclination (gradient) of the user's face, instead of the direction of the head. As a result, the proposed mechanism can discriminate between intentional and unintentional behavior, thereby preventing potential accidents, when the user instinctively turns their head to look at an obstacle. Furthermore, the proposed control mechanisms only require minimal user motion, making the system safer, more comfortable, and more adaptable to the severely disabled when compared to conventional methods.

**Figure 3:**
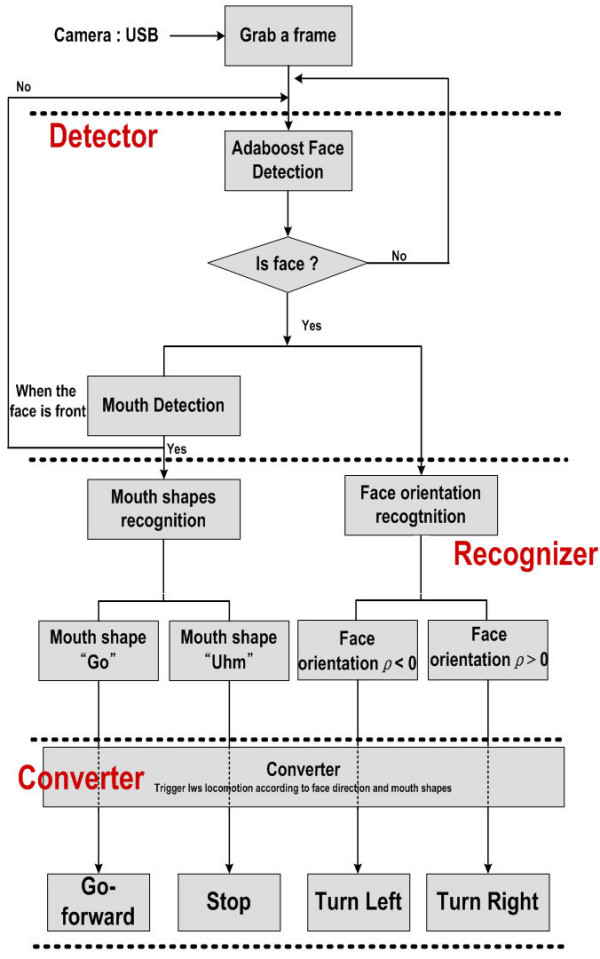
**The overall architecture of the proposed control system**.

Figure 3 describes the process to recognize user's gestures, where the recognition is performed by three steps: Detector, Recognizer, and Converter. First, the facial region is obtained using the Adaboost algorithm, and the mouth region is detected based on edge information. These detection results are then delivered to the Recognizer, which recognizes the face inclination and mouth shape using *K*-means clustering and a statistical analysis, respectively. Thereafter, the recognition results are delivered to the Converter, which operates the wheelchair. Moreover, to fully guarantee user safety 10 range sensors are used to detect obstacles in environment and avoid them. In what follows, the details for the respective components are shown.

### Facial Feature Detector: Detect User's Face and Mouth from PC Camera

For each frame of an input streaming, this module localizes the facial region and mouth region, and sends them to the Recognizer. The facial region is obtained using the Adaboost algorithm for robust face detection, and the mouth region is obtained using edge information within the facial region.

**Figure 4:**
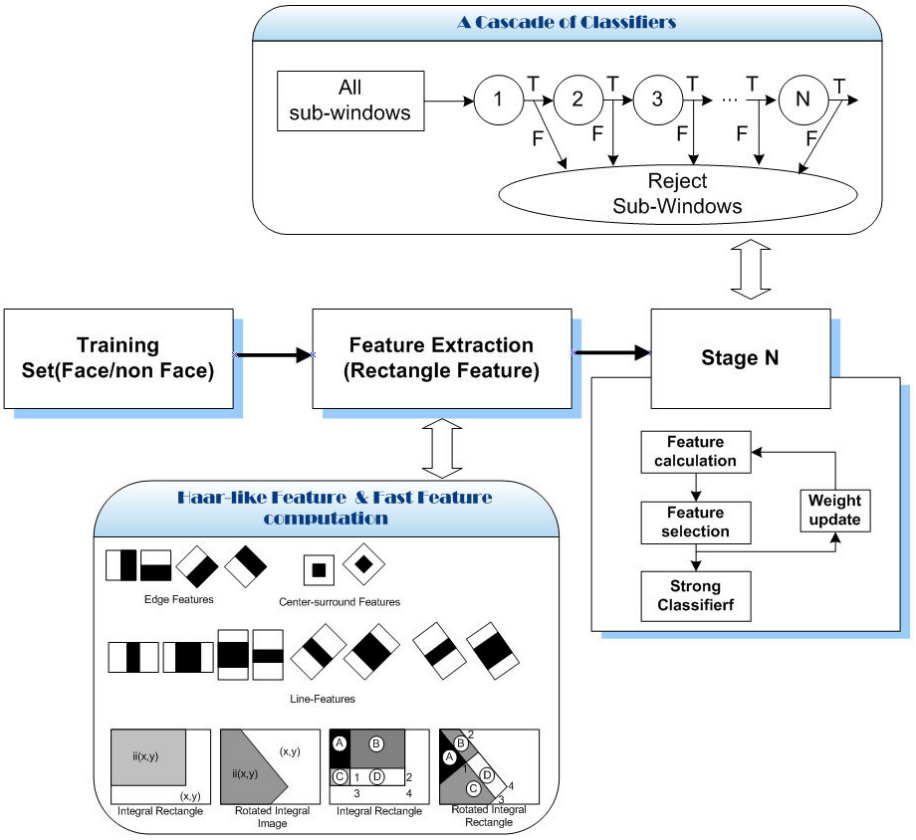
**Outline of face detection using Adaboost algorithm**.

For application in a real situation, the face detection should satisfy the following two requirements: 1) it should be robust to time-varying illumination and cluttered environments and 2) it should be fast enough to supply real-time processing. Thus, the Adaboost algorithm is used to detect the facial region. This algorithm was originally proposed by Viola and has been used by many researchers. The Adaboost learning method is an iterative procedure for selecting features and combining classifiers. For each iteration, the features with the minimum misclassification error are selected, and weak classifiers are trained based on the selected features. The Adaboost learning method keeps combining weak classifiers into a stronger one until it achieves a satisfying performance. To improve the detection speed, a cascade structure is adopted in each of the face detectors, to quickly discard the easy-to-classify non-faces. This process is illustrated in Figure 4.

**Figure 5:**
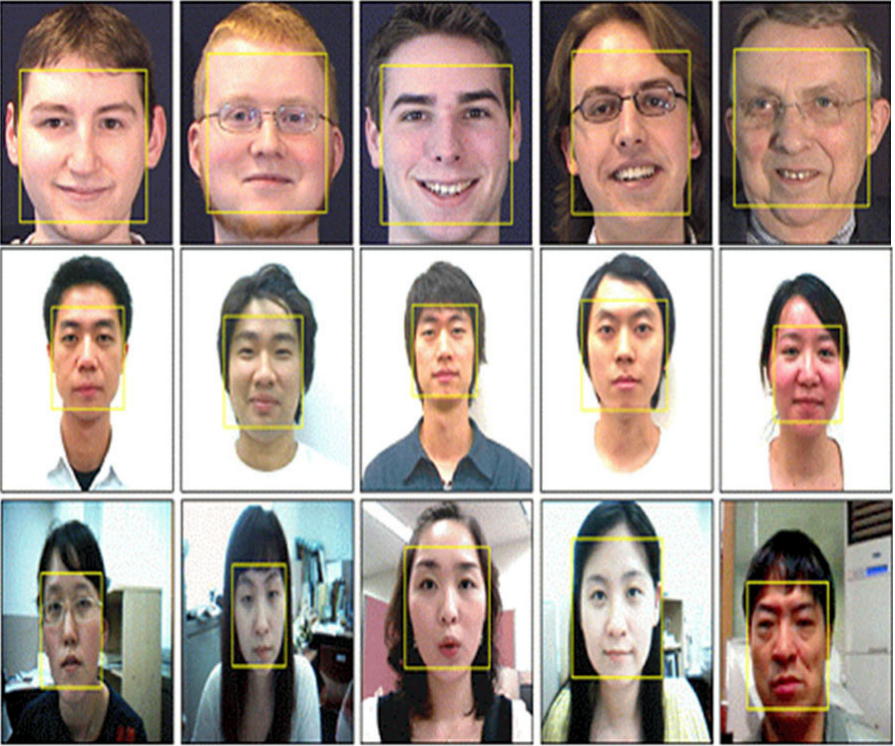
**Face Detection Results**. (a) the results for MMI DB, (b) the results for VAK DB, (c) the results for online streaming data.

Figure 5 shows some face detection results. To demonstrate its robustness, the face detection method was tested with several standard DBs such as VAK DB [20]. Moreover, it was tested on the data obtained from real environment. Figures 5(a) and 5(b) show the results for VAK DBs, respectively. And Figures 5(c) is the results for online streaming data. As seen in Figure 5, the proposed method is robust to the time-varying illumination and the cluttered environments.

To reduce the complexity of the mouth detection, it is detected based on the position of the facial region using the following properties: 1) the mouth is located in the lower region of the face and 2) the mouth has a high contrast compared to the surroundings. Thus, the mouth region is localized using an edge detector within a search region estimated using several heuristic rules based on the facial region. The details for the search region are given in our previous work by the current authors [21].

**Figure 6:**
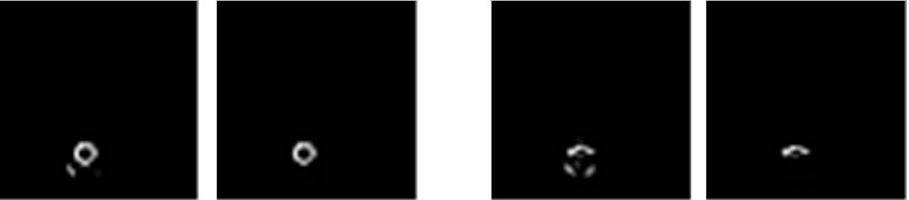
**The mouth detection results**. (a) edge detection results, (b) noise removed results.

Figure 6 shows mouth detection results. Since the detection results include both narrow edges and noise, the noise is eliminated using the post-processing.

### Facial Feature Recognizer: Recognize Face Inclination and Mouth Shape of the Intended User

This module recognizes the user's face inclination and mouth shape, both of which are continuously and accurately recognized using a statistical analysis and template matching. As a result, the proposed recognizer enables the user to control the wheelchair directly by changing their face inclination and mouth shape. For example, if the user wants the wheelchair to move forward, the user just says "Go." Conversely, if the user wants the wheelchair to stop, the user just says "Uhm." Here, these commands only have an effect when the user is looking forward, thereby preventing over-recognition when user is talking to someone. Plus the direction of the IW is determined by the inclination of the user's face instead of the direction of the user's head.

Let *ρ *denote the orientation of the facial region. Then, *ρ *can be calculated by finding the minimized inertia, which is defined as follows.

(1)

where the A is the number of pixels in the region R, and *d *is the distance between pixel (*r*, *c*) and axis of inertia which pass through the centroid, (, ). We obtain these properties by  and .

**Figure 7:**
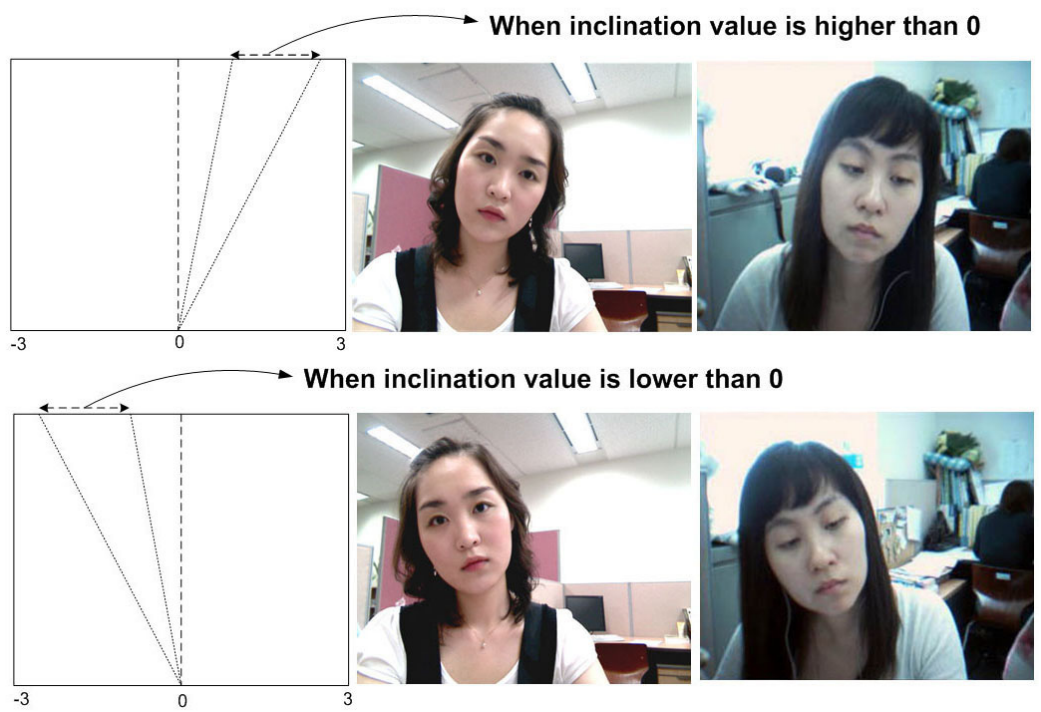
**The recognition results for face inclination**. (a) the commands of turn-left, (b) the commands of turn-right.

To minimize the inertia, the derivative is taken with respect to *β*. Accordingly, the orientation *ρ *can then be obtained by equation (2).

(2)

where *μ*_*rr*_, *μ*_*cc *_and *μ*_*rc *_are the second moments, the respective of which are defined as  and . If the value of ρ is less than 0, this means that the user nods their head slanting to the left. Otherwise, it means that the user nods their head slanting to the right. Figure 7 shows the recognition results for the face inclination.

**Figure 8:**
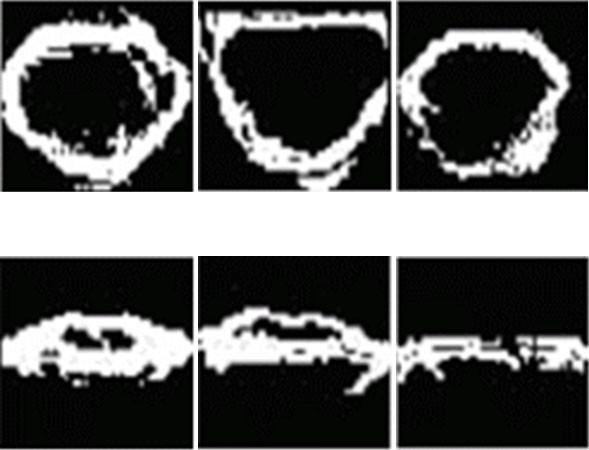
**The mouth shape templates**. (a) "Uhm" mouth shape templates and, (b) "Go" mouth shape templates.

To recognize the mouth shape in the current frame, template matching is performed, where the current mouth region is compared with mouth-shape templates. These templates are obtained by *K*-means clustering from 114 mouth images. *K*-means clustering is a method of classifying a given data set into a certain number of clusters fixed a priori. In this experiment, multiple mouth-shape templates were obtained, which consisted of 6 different shapes of "Go" and "Uhm." Figure 8 shows the mouth shape templates.

The results of the comparing the templates with a candidate are represented by matching scores. The matching score between a mouth-shape template and a candidate is calculated using the Hamming distance, where the Hamming distance between two binary strings is defined as the number of digits in which they differ.

Here the matching scores for all the mouth-shape templates and a mouth candidate are calculated, and the mouth-shape template with the best matching score is selected.

### Converter: Translate User's Gesture into IW's Control Commands

**Figure 9:**
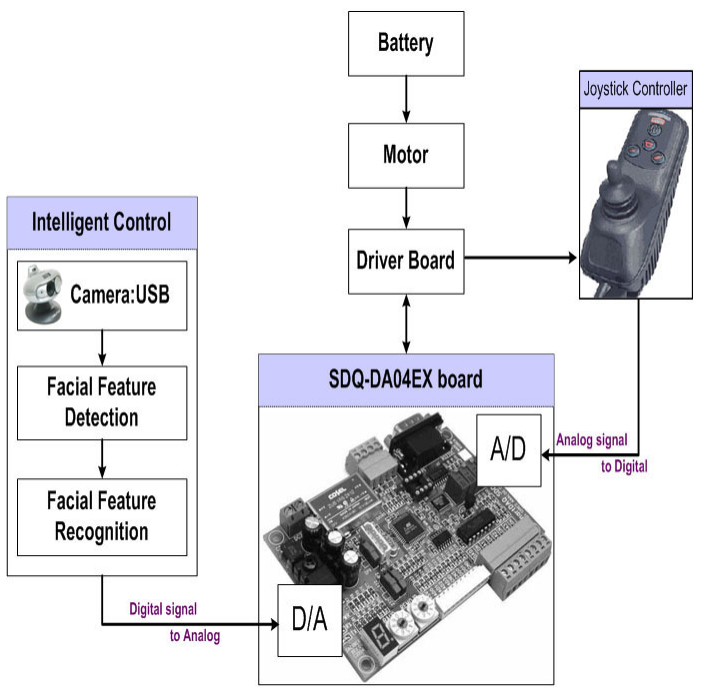
**Data Acquisition board (SDQ-DA04EX)**.

The proposed system uses a data acquisition board as a converter to translate the user's gestures into control commands for the IW. Similar to a general electric powered wheelchair, which is controlled by the voltage passed to the joystick, a data acquisition board (SDQ-DA04EX) is used to transform the ADC function and DAC. Figure 9 shows the data acquisition board used in our IW. The board is connected to a computer through a serial port and programmed using Visual Basic. The programmed function then translates the user's gestures into control commands for the IW.

The commands given from the user interface are passed to the control program running the wheelchair through the serial port. The board program then controls the speed and direction of wheelchair by modifying the voltage passing through the wheelchair.

Table 3 shows command map between wheelchair movement and output voltage. The proposed system is able to control both the direction and the velocity of the wheelchair, as the user can produce a different output voltage by changing their mouth shape or face orientation. In addition to simple commands, such as go-forward, go-backward, turn-left, or turn-right, the proposed system can also give a mixture of two simple commands, similar to joystick control. For example, the wheelchair can go in a 45 degree direction by combining the go-forward and go-right commands.

**Table 3:** Operation Volts of Intelligent Wheelchair

**Commands**	**Output 1**	**Output2**
**Go**	2.45 V~3.7	2.45 V

**Back**	1.2 V~2.45 V	2.45 V

**Left**	2.45 V	1.2 V~3.7 V

**Right**	2.45 V	2.45 V~3.7 V

**Stop**	2.45 V	2.45 V

## Results

### Experimental Environments

The interface system of IW was developed in PC platform: the operation system is Windows XP and CPU is Pentium 1.7 GHz. The Camera is Logitech, which was connected to the computer using the USB port and supplied 30 color images sized at 320 × 240 per second.

To assess the validity of the proposed system, it was tested on 34 participants, including 17 disabled and 17 able bodied users. The disabled users had the following disabilities: ataxia and quadriplegia from cord-injuries. The details are summarized in Table 4.

**Table 4:** Testing Groups

**Stage Time (.ms)**	**Number**	**EPW usage**	Computer usage(*)(%)
**Able-bodied users**	17	0%	100%(92%)

**Disabled users**	17	81%	64%(23%)

The experiments were performed by two steps. First, the performance of the proposed system is presented, which was tested in various environments. The effectiveness of the proposed system is then discussed in comparison with other methods.

### Experiment I: To measure the accuracy of our interface

**Figure 10:**
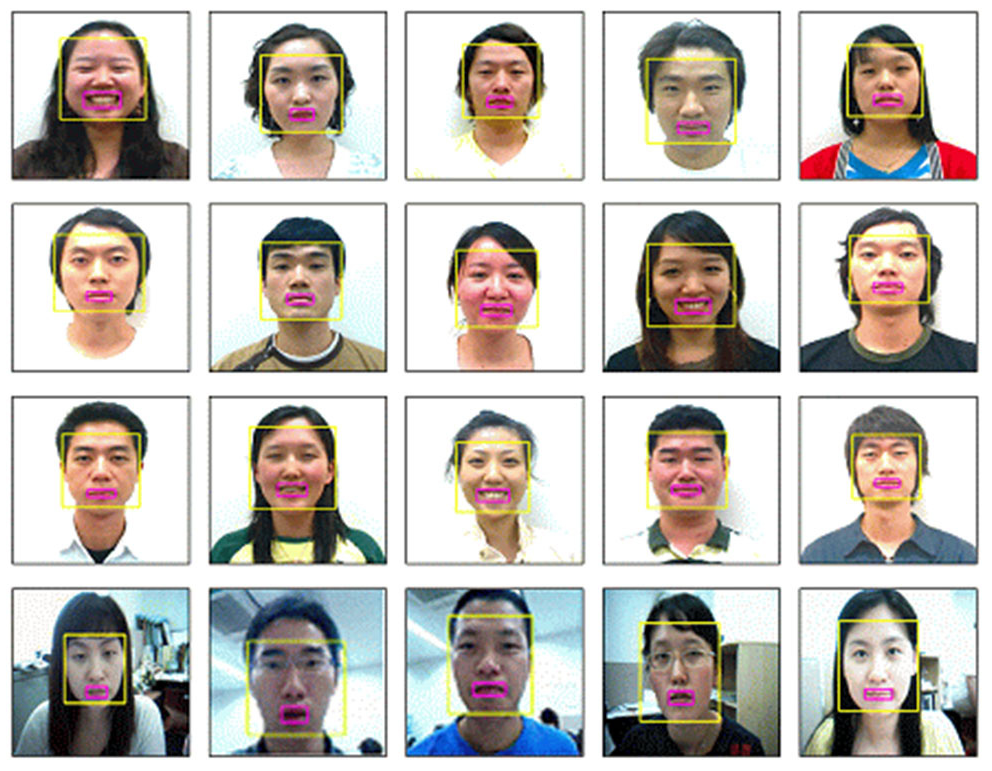
**Face and mouth detection results**.

For the proposed system to be practical in the real environments, it should be robust to various illuminations and cluttered backgrounds. Thus, the proposed method was applied to detect and recognize the user's facial features in a complex background. Figure 10 shows that the facial feature detection results. The scenes had a cluttered stationary background with a varying illumination. As seen in Figure 10, the results accurately detected the face and mouth, confirming the robustness to time-varying illumination, and low sensitivity to a cluttered environment.

**Figure 11:**
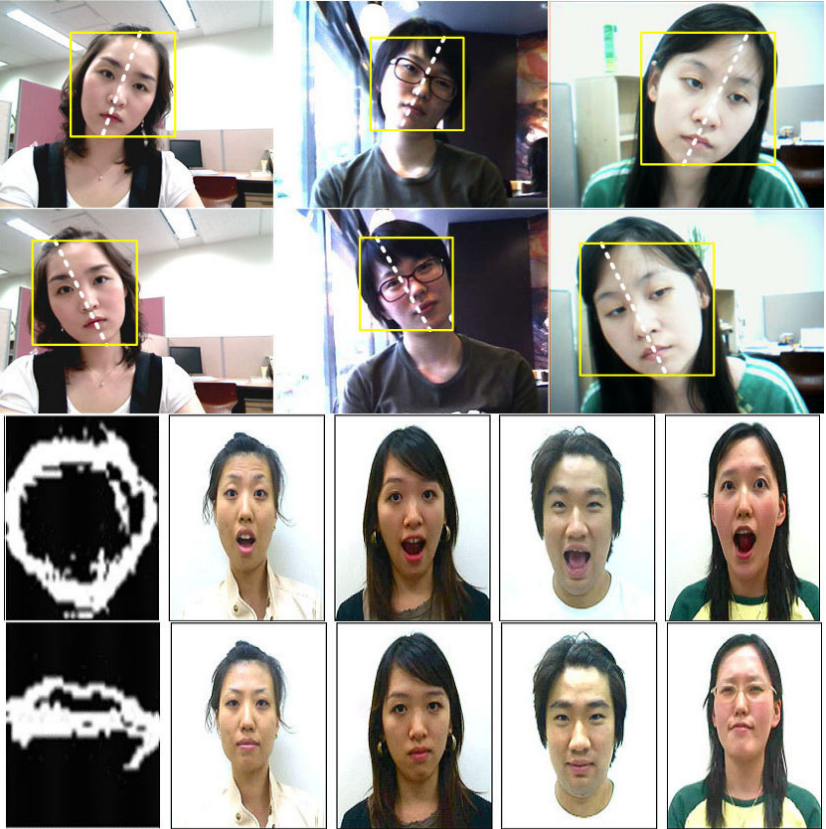
**Face and mouth recognition results**. (a) face inclination recognitions, (b) mouth shape recognitions.

With the proposed system the user's intention is represented by the inclination of the face and shape of the mouth, making the accurate recognition of these features crucial. Figures 11(a) and 11(b) show the recognition results for the face inclination and the mouth shapes, respectively. As shown in these figures, they are continuously and accurately recognized.

To quantitatively evaluate the performance of the proposed system, we asked each user to perform contain commands, such as go straight, stop, turn left or turn right and repeat the action 50 times.

Table 5 shows the average time taken to detect the face and facial features, then recognize them in an indoor and outdoor environment. As a result, the average time taken to process a frame was about 62 ms, allowing the proposed system to process more than 15 frames/sec on average (16 frames/sec in indoor and 14 frames/sec in outdoor). Table 6 shows the recognition rates of the proposed interface for the respective commands. The proposed system shows the precision of 100% and the recall of 96.5% on average. Thus, this experiments proved that the proposed system can accurately recognize user's intentions in real-time.

**Table 5:** Processing Time (.ms)

**Stage**	**Indoor**	**Outdoor**
**Face Detection**	30	32

**Mouth detection**	15	18

**Face inclination recognition**	2	2

**Mouth shape recognition**	15	16

**Total**	62	68

**Table 6:** Performance Evaluation Results

**Commands**	**Recall**	**Precision**
**Left turn**	0.98	1

**Right turn**	0.94	1

**Go straight**	0.96	1

**Stop**	0.98	1

**Figure 12:**
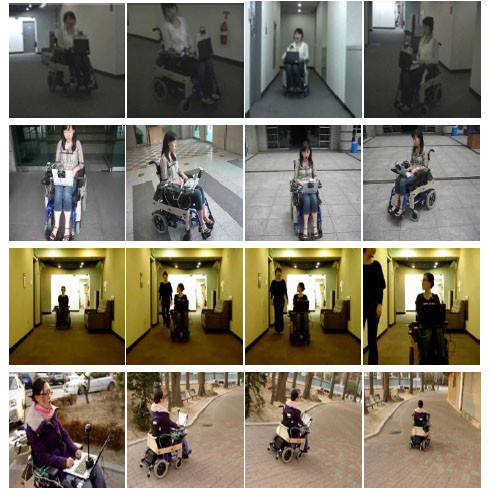
**IW controls on real environments**.

Figure 12 shows some snapshots for the proposed system to be applied on various environments. Outdoor environments have a time-varying illumination and more complex background, as shown in Figures 12(b) and 12(d). However, despite those complexities, the proposed system worked very well in both environments. In particular, in case of someone comes to talk the user (in Figure 12(c)), our system can accurately discriminate between intentional and unintentional behaviors, thereby preventing potential accidents, when the user instinctively turns their head to look at a person.

### Experiment II: To compare with other interfaces

To prove the efficiency and effectiveness of the proposed IW interface, it was also compared with other systems. Here, two methods were adopted, one is headband-based method and the other is method using face tracking [22,23]. Then, the former belongs to the intrusive method and the latter belongs to the vision-based method. In the headband-based method, the go-and-stopping is controlled by nodding user's head to the front or to the rear, and the direction is changed by nodding user's head to the left side or to the right side. In such system, the head motions are measured through a headband that includes an accelerometer sensor. On the other hand, a face-based interface detects user's face in the first frame and tracks it continuously, and then user's face are detected using skin-color model.

**Figure 13:**
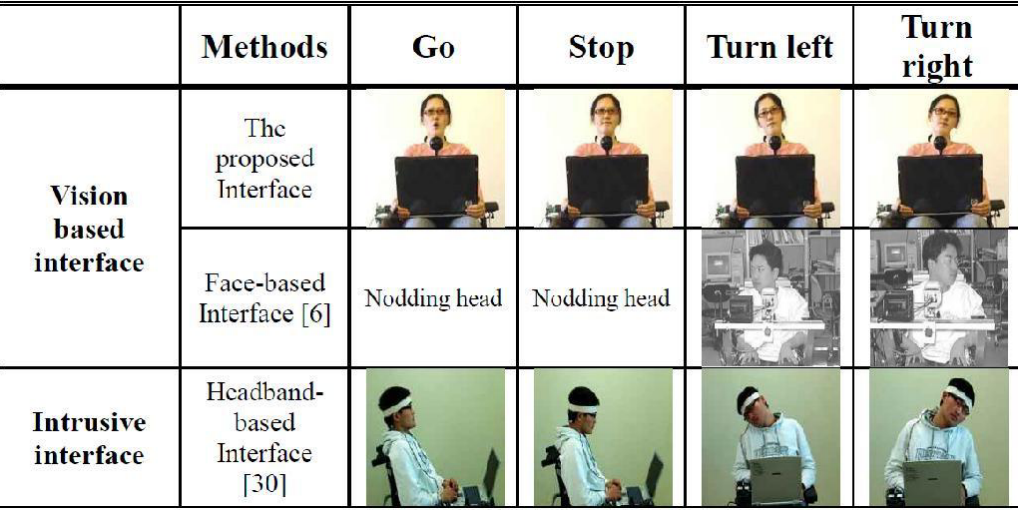
**Intelligent Wheelchair input methods**.

Figure 13 shows the control commands for respective methods. When visually inspected, our system requires the smaller motions than others. This tells us our system is more comfortable and suitable to the severely disabled.

**Figure 14:**
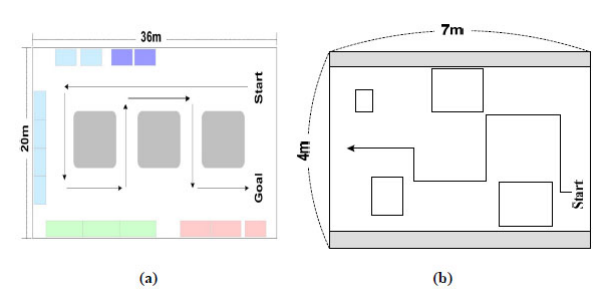
**Some examples of test maps**. (a) Outdoor test map, (b) indoor test map.

For the practical use by the severely disabled, such systems should be operable on both indoor and outdoor environments. Thus, the three systems were evaluated across indoors and outdoors, changes in time of day and weather conditions. Such conditions are summarized in Table 7. And some test maps in indoor and outdoor environments are shown in Figure 14.

**Table 7:** Test environments

**Places**	**Time and illumination**
**Indoor**	(Daytime, fixed illumination)
	
	(Nighttime, fixed illumination)

**Outdoor**	(Daytime, time-varying illumination and a shadow)
	
	(Nighttime, -)

**Indoor to outdoor, or vice versa**	(Daytime, time-varying illumination and shadow)

We asked the participants to navigate each map 10 times using three interfaces. The performances for three interfaces were then evaluated in terms of the accuracy and speed. In those experiments, the face-based method was tested in only the indoor environments, due to its sensitivity to the time-varying illumination. As mentioned above, it used the skin-color model to extract user's face, so it is very sensitive to illumination changes.

Tables 8 and 9 show the summarized performance comparisons for the three methods. Table 8 shows the average times taken to reach the destination for three interfaces. Among three methods, the face-based method is the slowest, whereas there is no significant difference between our method and the head-band method. Meanwhile, Table 9 shows the recognition accuracies of three methods, where the proposed-based method produced the best performance with an average accuracy of 96.5%, while the face-based method had an accuracy of 87.5% and the headband-based method had an accuracy of 88%. In experiments, the face and headband based methods made over-recognition or miss-recognition in some environments. When travelling on an uphill (or downhill) place, the headband method often missed the go-straight and stop commands. And the face-based method often missed user's face so that it cannot track user's gestures. Moreover, it cannot discriminate between intentional and unintentional behaviors, thereby making potential accidents, when the user instinctively turns their head to look at an obstacle.

**Table 8:** Processing Time

**Methods**	**Average time taken to reach the destination (.ms)**
**Proposed method (test in indoor and outdoor)**	48.31 s

**Headband-based method (test in indoor and outdoor)**	48.61 s

**Face-based method (test in indoor)**	51.23 s

**Table 9:** Accuracy (%)

**Methods**	**Precision**	**Recall**
**Proposed method (test in indoor and outdoor)**	100	96.5

**Headband-based method (test in indoor and outdoor)**	89	88

**Face-based method (test in indoor)**	87	87.5

Consequently, these comparisons proved the efficiency and effectiveness of the proposed system. Moreover, as shown in Figure 13, the proposed method requires more minimal user motion than the head-band method, making the proposed system more suitable for the severely disabled than conventional methods.

## Discussion & conclusion

In this paper, we developed an IW system, which is adapted, efficient, and robust for the disabled people with physical disabilities. In the proposed system, the direction of IW is determined by user's face inclination, while its going and stopping is determined by user's mouth shape. The advantages of the proposed system include the followings: 1) minimal user motion, making the proposed system more adaptable to the severely disabled, than conventional methods. 2) robustness to a cluttered background and the time-varying illumination and 3) accurate recognition of user intention based on discriminating between intentional and unintentional behavior. To prove these advantages, the proposed system was tested with 34 users on the indoor outdoor environments.

However, to guarantee full user safety, the proposed system also needs to be able to detect and avoid obstacles automatically thus further research in this area is currently underway.

## Competing interests

Konkuk Univ. will apply for a patent for the proposed IW interface. The authors are composed of two PhD. Candidates and a professor. The affiliated Konkuk Univ. is the educational institute, so we are not participating in any interests.

## Authors' contributions

JJ (Ju, Jin Sun), SY (Shin, Yunhee), KE (Kim, Eun Yi) conceived of the research and participated in its design and coordination. JJ implemented the facial feature detection and recognition software, conducted the user trials and drafted the manuscript. SY implemented the hardware for the IW, and interfaced the vision system with the DAQ board. KE reconstructed the proposed system to complete additional user testing and revised the manuscript. All authors read and approved the final manuscript.
